# Recent Progress in the Development of Liver Fluke and Blood Fluke Vaccines

**DOI:** 10.3390/vaccines8030553

**Published:** 2020-09-22

**Authors:** Donald P. McManus

**Affiliations:** Molecular Parasitology Laboratory, Infectious Diseases Program, QIMR Berghofer Medical Research Institute, Brisbane 4006, Australia; Don.McManus@qimrberghofer.edu.au; Tel.: +61-(41)-8744006

**Keywords:** *Fasciola*, *Opisthorchis*, *Clonorchis*, *Schistosoma*, fasciolosis, opisthorchiasis, clonorchiasis, schistosomiasis, vaccine, vaccination

## Abstract

Liver flukes (*Fasciola* spp., *Opisthorchis* spp., *Clonorchis sinensis*) and blood flukes (*Schistosoma* spp.) are parasitic helminths causing neglected tropical diseases that result in substantial morbidity afflicting millions globally. Affecting the world’s poorest people, fasciolosis, opisthorchiasis, clonorchiasis and schistosomiasis cause severe disability; hinder growth, productivity and cognitive development; and can end in death. Children are often disproportionately affected. *F. hepatica* and *F. gigantica* are also the most important trematode flukes parasitising ruminants and cause substantial economic losses annually. Mass drug administration (MDA) programs for the control of these liver and blood fluke infections are in place in a number of countries but treatment coverage is often low, re-infection rates are high and drug compliance and effectiveness can vary. Furthermore, the spectre of drug resistance is ever-present, so MDA is not effective or sustainable long term. Vaccination would provide an invaluable tool to achieve lasting control leading to elimination. This review summarises the status currently of vaccine development, identifies some of the major scientific targets for progression and briefly discusses future innovations that may provide effective protective immunity against these helminth parasites and the diseases they cause.

## 1. Introduction

This article provides an overview of recent progress in the development of vaccines against digenetic trematodes which parasitise the liver (*Fasciola hepatica*, *F. gigantica*, *Opisthorchis* spp., *Clonorchis* spp.) and blood system (*Schistosoma* spp.) and are the cause of important but neglected tropical diseases affecting millions. *F. hepatica* and *F. gigantica* are also important parasites of domesticated animals, greatly affecting the economy of the agricultural community worldwide [1]. Because of their relatedness and life cycle similarities [1], there is a clear parallel in the approaches taken towards vaccine development (e.g., methods used in the identification of candidate antigens, the technology applied to produce vaccines and their testing in mammalian hosts) for each of these fluke species. Current progress and recent innovations that may deliver vaccines against these parasites in the future are considered and emphasised. However, it is a sobering and sad reality that, notwithstanding the considerable efforts undertaken, and despite encouraging developments recently in some areas, no licensed products are available currently for the prevention of any of the infections caused by these parasitic worms. The realisation that, as yet, there is no commercial vaccine available against any of member of this important group of parasites underscores the need for continued, indeed increased, efforts towards achieving this elusive goal.

## 2. Fasciolosis 

Fasciolosis is a parasitic zoonosis resulting from exposure to *Fasciola hepatica* or *F. gigantica* liver flukes. Human fascioliasis generally results from the consumption of moistened or semiaquatic vegetation (water chestnuts, watercress, grass and salad vegetables) to which the infective metacercaria stage attaches. Sheep, pigs, cattle and other domesticated herbivores act as definitive hosts from which *Fasciola* eggs are released, hatch in water to form ciliated free-swimming miracidia that infect lymnaeid snails which act as intermediate hosts. The infected molluscs release cercariae that encyst on vegetation and freshwater plants to form the metacercarial cysts. After ingestion, the metacercariae excyst and the immature flukes, termed newly excysted juveniles (NEJ), emerge from the cysts in the intestine and migrate through the intestinal wall into the peritoneal cavity. These travel through the hepatic parenchyma, causing extensive tissue damage resulting in necrosis and eosinophilic infiltration as the parasites gain access to the bile ducts where they mature into adult worms. Their presence in the bile ducts leads to the proliferation of ductal epithelium, inflammation and fibrosis; heavy infections can result in severe liver damage and cirrhosis. The adult hermaphroditic flukes release eggs into the hepatic and common bile duct of the host which exit in stool.

Fasciolosis is endemic throughout the world infecting 600 million domestic ruminants, causing major economic losses estimated to be US$3 billion per annum; some 17 million people are also infected in 61 countries with 180 million at risk of infection and the burden of disease due to fasciolosis is estimated at 90,000 disability-adjusted life years [1,2,3,4]. Regions of high endemicity are in Bolivia and Peru where *F. hepatica* is the causative species. *Fasciola* species appear insensitive to praziquantel treatment and triclabendazole, a benzimidazole derivative, is the drug of choice; alarmingly, however, there are reports of parasite isolates in livestock that are insensitive to this drug [4]. Furthermore, cross-fertilisation between *F. hepatica* and *F. gigantica* occurs and this can result in more virulent and pathogenic hybrids [4]. Clearly the development of an effective vaccine is paramount and would represent the most appropriate and sustainable path for the control of fascioliasis.

### Fasciolosis Vaccines: An Update 

There have been considerable advances in identifying potential vaccine molecules for the control of fasciolosis in livestock, but it is fair to say that the level of efficacy required for commercialisation has not yet been reached. A major issue to overcome is the immune suppression/modulation by fasciolids that prevents the development of protective T helper (Th)1 immune responses, evidenced by the lack of immunity observed in naturally and experimentally infected animals [4,5,6,7,8,9,10,11,12]. This is due to the development of a strong regulatory/Th2 type immune response during active infection that potently suppresses the host’s Th1 response [4]. Nevertheless, a wealth of studies provided evidence that acquired immunity occurs following *Fasciola* infection in animals, and protection against *F. hepatica* is inducible in rats, sheep or cattle by passive transfer of immune sera and cells [13]. The challenge is to identify the specific antigens that are the targets of this protective immunity and incorporate these in vaccine formulations that induce Type 1 responses to enhance vaccine efficacy [14].

Approaches used to date to identify vaccine candidates for *F. hepatica* and *F. gigantica* include: (I) analysis of antigens that cross-react with sera from animals with other trematode infections; (II) orthologous antigens identified as vaccine candidates in other species; and (III) rational selection of antigens pivotal in liver fluke survival [14]. Many vaccine studies in livestock have evaluated the value of these candidate antigens, and Table 1 summarises the level of protective efficacy observed with some single or combination experimental *F. hepatica* vaccines [15]. Results of a compilation of additional efficacy trials, over the past three decades, with vaccines for both *F. hepatica* and *F. gigantica* tested in livestock, are also available [14]. Lead candidates tested include fatty acid binding proteins (FABP), glutathione *S*-transferase (GST), cathepsin L1 (CatL1), peroxiredoxin (Prx) and the gut-associated exopeptidase leucine aminopeptidase (LAP). A feature of these studies was the high level of variability in vaccine efficacy that was evident between trials, which may have resulted from differences in the antigen source, the adjuvant used and the host species vaccinated; variation between animals from the same or different breeds was also commonly observed [5,14].

The genomes of *F. hepatica* (size: 1.14–1.3 Gb) and *F. gigantica* (size: 1.04–1.13 Gb) exhibit a high degree of genomic polymorphism [16,17,18], and it has been speculated that the variability in vaccine efficacy widely reported could be attributed, at least in part, to phenotypic differences resulting from the variation in sequences of the vaccine candidates amongst parasites used in each challenge infection [15]. Indeed, antigenic polymorphism has long been considered an important mechanism of immune evasion used by a number of pathogens, including helminths [19]. However, genetic analysis of several vaccine candidates (FhCL1, FhCL2, FhPrx (peroxiredoxin), FhLAP and FhHDM (*F. hepatica* helminth defence molecule [8])) showed that *F. hepatica* exhibits only a low level of allelic variability in the sequences encoding each of these proteins amongst isolates from different geographical regions; this suggests the variability reported among vaccine studies are not related to heterogeneity in these genes, thereby reinforcing the idea that they would be suitable immunogens against liver fluke parasites globally [15]. Further elaboration of host-associated effects and the level of animal susceptibility to infection may be able to explain the variability observed in *F. hepatica* vaccine trials.

As with other anti-trematode vaccines, adjuvant choice is a central issue. There is a basic requirement to discover an adjuvant that is as potent as Freund’s Complete Adjuvant that does not produce side effects but is capable of generating strong and effective protective Th1 immune responses in a broad spectrum of hosts to overcome animal variability [15]. Furthermore, native fluke antigens appear, in general, to promote more effective immune responses than recombinant vaccines, inferring that post-translational modifications (phosphorylation, glycosylation, ubiquitination, nitrosylation, methylation, acetylation, lipidation and proteolysis) may be critical in generating the sufficiently robust levels of protective immunity required, an important factor that will need consideration in the future [15]. It is possible that the key molecules that stimulate the required level of protective immunity have not yet been characterised. As such, future vaccine discovery may need to specifically target fasciolid molecules such as surface tegumental proteins/glycoproteins and secreted components, including surface molecules of secreted extracellular vesicles that are involved in early stage migration in the host, in tissue penetration and in immunomodulation [5,20,21]. Moreover, the identification of antigens shared between *F. hepatica* and *F. gigantica* can provide an avenue for possible development of a dual purpose and cost-friendly vaccine against both liver fluke species [22]. The availability of substantial data on the transcriptomes of liver fluke species can aid in this process as well as in the identification of new vaccine targets [23,24,25,26,27]. It is important to consider that mathematical modelling shows that fasciolid vaccines, even if they are unable to provide complete protection, could contribute markedly towards fluke control and, at the same time, delay the spread of anthelmintic resistance through the reduced use of triclabendazole [28].

## 3. Opisthorchiasis

The food-borne liver fluke *Opisthorchis viverrini* is a parasite of fish-eating mammals, including humans, and infects 10 million people in different countries of Southeast Asia including Thailand, Cambodia, Lao PDR, Vietnam and Myanmar [29,30,31]. The related species, *O. felineus*, is endemic mainly in Europe and Russia and infects 2 million individuals [32]. Felines are important reservoir hosts in endemic areas for both *O. viverrini* and *O. felineus* [29,30,31,32]. The hermaphrodite adult worms live in the bile ducts, where they cause liver pathology, including malignant cholangiocarcinoma (bile duct cancer) (CCA) [33,34]. Eggs produced by the adult worms pass down the bile ducts to the intestine and out with the faeces. In water, snails of a number of species (*Bithynia*, *Parafossarulus* and *Thiara*) serve as the first intermediate host which ingest an egg that hatches to release the miracidium. Sporocyst and redia generations occur and cercariae are produced; these leave the snail and encyst as metacercariae in the muscle of freshwater cyprinid fish. With both *O. viverrini* and *O. felineus,* people are infected through eating raw or undercooked infected fish. Current control efforts rely on health education and drug treatment measures that are, in the long term, unsustainable in isolation; praziquantel is the only drug routinely employed against the *Opisthorchis* parasites and there is no effective drug against CCA [35,36]. A vaccine would reduce parasite burden, confer anti-cancer protection and help facilitate the control and possible elimination of opisthorchiasis.

### Opisthorchiasis Vaccines: An Update

A recent review by Sripa et al. [37] provides an in-depth discussion of immune responses to opisthorchiasis. Following infection, adaptive (humoral and cell-mediated) and innate immune responses are stimulated by excretory/secretory (ES) products released by the liver fluke and by the resulting tissue damage; however, there appears to be only partial protection conferred in the hamster experimental model of opisthorchiasis and in humans; this may possibly be due to immune evasion mechanisms of the parasite, immune suppression, the worm tegument able to withstand immune attack and/or the location of the parasite within the bile ducts [37]. This limited level of natural protective immunity generated will make the development of a vaccine against opisthorchiasis challenging. Nevertheless, the availability of such an intervention is feasible and is urgently needed to serve the dual purpose of potentially reducing both parasite burdens and the incidence of CCA.

Data from recent omics investigations on *O. viverrini* and *O. felineus* provide the foundation for identifying targets leading to the discovery of new interventions, including vaccines, against opisthorchiasis and opisthorchiasis-induced bile duct cancer. An extensive overview of molecular research on *Opisthorchis* and opisthorchiasis is available [38]. Draft genomes are published for both *O. viverrini* (634.5 Mb) [39] and *O. felineus* (684 Mb) [40]. Furthermore, there is a wealth of available data on the transcriptome/secretome and proteome of both species providing key information that can be used for identifying potential novel antigens for vaccine development [24,40,41,42,43,44,45,46,47]. Presumptive anti-worm and anti-fecundity vaccine candidates include glutathione S-transferase, other anti-oxidant/detoxification proteins, fatty acid binding protein, myoglobin, ferritin, various proteases (e.g., cathepsin F, B1), eggshell protein, SH3-domain containing protein, Ly6-domain containing protein, paramyosin, calreticulin and granulin-like growth factor (Ov-GRN-1) [41,44,48]; however, none appear to have been tested in the hamster vaccination model. Successful vaccination of hamsters has, nevertheless, been reported using *O. viverrini* exosome-like extracellular vesicles (EVs) and recombinant tetraspanin proteins (TSPs) derived from the surface of EVs; generated antibody responses blocked EV uptake by target bile duct cells and the vaccines resulted in partial levels of protective efficacy against *O. viverrini* challenge [49]. The same group obtained similar levels of protective efficacy in hamsters vaccinated with recombinant protein encoding for the large extracellular loop of *O. viverrini* tetraspanin-2 expressed in *Pichia pastoris* [50]. Partial protection was also achieved using a chimeric tetraspanin-leucine aminopeptidase subunit vaccine against *O. viverrini* infection in the hamster vaccine challenge model [51].

## 4. Clonorchiasis

Clonorchiasis, caused by *Clonorchis sinensis* (also known as the Chinese or oriental liver fluke), is an important food-borne parasitic disease of the liver and one of the most common of the neglected tropical diseases zoonoses [1,52,53,54,55]. *C. sinensis* belongs to the same family (Opisthochiidae) as the *Opisthorchis* spp., and the life cycle and aetiology are very similar [1,54]. The adult worms inhabit the bile ducts, and *C. sinensis* infection is closely associated with cholangiocarcinoma (CCA), fibrosis and other human hepatobiliary diseases, making it a serious public health problem in areas where it is endemic [52,53,54,55]. The eggs of *C. sinensis* (like those of *Opisthorchis*), rarely among the trematodes, hatch only on ingestion by the snail intermediate host [52,53,54,55]. People who consume raw or undercooked fish are at risk for liver fluke infection. The fluke passes its life cycle in three different hosts, namely freshwater snails (e.g., *Parafosarulus* and *Alocinma*) as first intermediate hosts, freshwater fish (e.g., *Cyprinus carpio*, *Ctenopharyngodon idellus* and *Carassius carassius*), as second intermediate hosts and mammals as definitive hosts. The main non-human hosts are dogs, cats, pigs, rats and camels. Currently, more than 200 million people are at risk of *C. sinensis* infection, with over 15 million infected in China, Taiwan, Hong Kong, India, Vietnam, North and South Korea and Japan. Treatment relies on praziquantel but, as with other fluke infections, its main limitation is that it does not prevent reinfection [52,53,54,55].

### Clonorchiasis Vaccines: An Update

Various attempts have been made to develop effective vaccines for clonorchiasis but so far testing has only occurred in rodents, predominantly in the rat infection model. The differences in immune response and resistance to reinfection between human beings and rodents suggests we are likely a long way from developing an effective human vaccine for clonorchiasis [53]. As a result, no commercially produced or effective product is available for the treatment of *C. sinensis* infection in human or other mammalian hosts [52,53]. Nevertheless, an early study in rats indicated that the development of a vaccine was feasible in that animals orally challenged with irradiated metacercariae of *C. sinensis* generated resistance to infection, characterised by low worm recovery, high IgG antibody titre and elevated levels of the Th1 type cytokines, IFN-gamma and IL-2 [56]. Subsequently, much as a result of interrogating the 547–580 Mb genome and analysing the transcriptome of *C. sinensis* [57,58,59,60], some key biological molecules from the liver fluke, including tegumental molecules, excretory/secretory products and metabolic enzymes, have been considered as potential vaccine candidates [53,57]. Consequently, a series of vaccine/challenge studies in rats (Table 2) showed that intramuscular injection of DNA plasmids encoding enolase, paramyosin, fatty acid-binding protein and cysteine proteinase, and subcutaneous inoculation with recombinant proteins (paramyosin, cathepsin B cysteine protease 2 and 3, enolase, hexokinase, soinRho GTPase and 14-3-3 epsilon) resulted in reductions in worm numbers; however, none of the candidates achieved worm reduction rates exceeding 70% [52,53]. In another approach, significant *C. sinensis* worm reduction rates were obtained in rats vaccinated orally with *Bacillus subtilis* spores expressing either enolase or a 22.3 kDa tegumental protein of *C. sinensis* [52]. This outcome raised the possibility of using an alternative avenue to prevent transmission of clonorchiasis through the oral delivery of a *C. sinensis* vaccine antigen as a probiotic feed additive to achieve immune killing of cercariae/metacercariae in the second intermediate, freshwater fish host [55]. Indeed, an oral vaccine based on *B. subtilis* spores expressing *C. sinensis* paramyosin protects grass carp (*Ctenopharyngodon idella*) from infection with cercariae [61].

## 5. Schistosomiasis

Schistosomiasis (also called bilharzia) is a neglected tropical disease caused by blood flukes of the genus *Schistosoma*. Six species commonly infect humans, of which three (*Schistosoma haematobium*, *S. mansoni* and *S. japonicum)* contribute the most human cases. Infective cercarial larvae develop in, and are released from, specific freshwater snail intermediate hosts (*Bulinus, Biomphalaria* and *Oncomelania*) before penetrating the skin of the definitive human host. Mature adult male and female worms reside in the pelvic (*S. haematobium*) or mesenteric (*S. mansoni* and *S. japonicum*) veins, where the females lay eggs released to the external environment in stool or urine. Eggs trapped in the surrounding tissues and organs, such as the liver, intestine and bladder, cause inflammatory immune responses (including granulomas) that result in intestinal hepato-splenic or urogenital disease. Schistosomiasis causes substantial morbidity in parts of the Middle East, South America, Southeast Asia and, particularly, in Sub-Saharan Africa. Globally, an estimated 779 million people are at risk of infection. Over 250 million people have infections with *Schistosoma* spp., with 201.5 million living in Africa. In relation to treatment, the anti-schistosomal drug praziquantel is safe and efficacious against adult worms of all the schistosome species infecting humans. Indeed, global community-based schistosomiasis control programmes, focusing on mass drug administration with praziquantel, are the current cornerstone of control efforts and are important to reduce morbidity. However, the drug does not prevent reinfection and the emergence of praziquantel drug resistance is a constant concern. The availability and deployment of an effective schistosomiasis vaccine, probably in combination with MDA and other interventions, may be the best hope for achieving extensive control and eventual elimination, a scenario supported by mathematical modelling. In 2016, *Science* ranked the schistosomiasis vaccine as one of the top 10 vaccines that needs to be urgently developed based on necessity and feasibility.

Two recent reviews on schistosomiasis systematically covered schistosome biology, genomics, proteomics and transcriptomics, clinical features, pathophysiology, disease progression, immunology and host-parasite interplay, epidemiology, diagnosis and management and control, including vaccination [62,63]. Some of the major recent advances in schistosomiasis vaccine development, emphasising the status of vaccines at different phases of human clinical trialling, are revisited and briefly summarised here. In addition, the first results of a recently established controlled human challenge infection model for clinically testing schistosomiasis vaccines are considered [64].

### 5.1. Schistosomiasis Vaccines: An Update

In the past several decades, over 100 schistosome antigens have been advanced as possible vaccines [63,65], and recently developed approaches provide an avenue to identify and test additional candidate molecules for protective efficacy [66,67,68]. Indeed, many proteins are exposed at, or released from, the parasite–host interface so many other possibilities remain for potential vaccine exploitation. Progress in the development and testing of a transmission blocking vaccine for zoonotic schistosomiasis japonica is not considered further here as the area has been recently extensively reviewed [65,69]. Four lead *Schistosoma* vaccines (Sh28GST, Sm-14, Sm-TSP-2 and Sm-P80), currently at differing phases of human clinical development [70,71,72], are now described.

#### 5.1.1. Sh28GST

Recombinant 28-kDa glutathione S-transferase of *S. haematobium* (rSh28GST), adsorbed to Alhydrogel (named Bilhvax), was shown in a Phase 1 clinical trial to be immunogenic, generating a Th2-type response in young healthy Caucasian male adults, and was well tolerated [73]. A subsequent Phase 2 clinical trial in Senegal showed that the Bilhvax vaccine combined with praziquantel treatment was safe in *S. haematobium* infected children and adults [74]. Results of a three-year Phase 3 trial in Senegalese children showed the Bilhvax vaccine was again well tolerated and immunogenic, characterised by antibodies able to inhibit 28 GST enzymatic activity, but an insufficient level of protective efficacy was reached at trial endpoint [74]. This lack of required efficacy may have been due to interference by individual praziquantel treatments administered each time a child was found infected as a safety measure; alternatively, the chosen vaccination regimen favoured the generation of blocking IgG4 rather than protective IgG3 antibodies [74]. Modifying the trial design, employing a different adjuvant to provide a more balanced isotypic response, and reducing the number of vaccine injections may improve the vaccine efficacy of rSh28GST in future clinical testing [74].

#### 5.1.2. Sm14

As with the *S. mansoni* homologue of Sh28GST (Sm28GST), Sm14 (14-kDa cytoplasmic fatty acid binding protein) was one of a group of potential *S. mansoni* vaccine candidates promoted in the late 1980s by TDR, the Special Programme for Research and Training in Tropical Diseases co-sponsored by the United Nations Children’s Fund (UNICEF), the United Nations Development Programme (UNDP), the World Bank and World Health Organization (WHO). The safety and immunogenicity of the recombinant Sm14 vaccine were evaluated in a Phase 1a clinical trial at a single site in Brazil in male volunteers from a non-endemic area for schistosomiasis [75,76]. Formulated with glucopyranosyl lipid A (GLA) adjuvant in an oil-in-water emulsion (SE) (GLA-SE), yeast-expressed rSm14 produced no serious adverse events; the Sm14/GLA-SE vaccine proved immunogenic, but no potentially deleterious specific IgE responses were generated, a positive outcome of the trial. A Phase 1b clinical study, evaluating the safety/immunogenicity of the Sm14/GLA-SE vaccine in healthy female volunteers, was concluded successfully in 2012 [76]. This paved the way for a Phase 2a clinical trial (https://clinicaltrials.gov/ct2/show/NCT03041766) undertaken during 2015–2017 in male adults resident in an area in the Senegal River Basin highly endemic for both *S. mansoni* and *S. haematobium*; the results of the trial confirmed the safety and long-lasting immunogenicity of the Sm14/GLA-SE vaccine [77]. A subsequent Phase 2b trial design and protocol were defined for infected Senegalese school children living in the same area endemic for both schistosome species and further Phase 2c and 2d (in Brazil) and Phase 3 (in Senegal) clinical trials with Sm14/GLA-SE are scheduled [77].

#### 5.1.3. Sm-TSP-2

Abundantly expressed on the outer tegumental membrane of schistosomes, tetraspanins (TSP) are a family of proteins continually exposed to the host immune system [78]. Required for tegument biogenesis and integrity, Sm-TSP-1 and Sm-TSP-2 are the main *S. mansoni* tetraspanins and are involved in tegument biogenesis and integrity [78]. Sm-TSP-2 conferred a significant level of protection in the mouse vaccine/challenge model; in addition, TSP-2 IgG1 and IgG3 antibodies were shown to positively correlate with protection in naturally immune people, whereas TSP-1 IgG antibodies did not [70,78]. Consequently, the Sm-TSP-2 antigen is considered a leading schistosomiasis vaccine candidate. It is being developed as a yeast-expressed and purified 9 kDa (extracellular domain) recombinant protein formulated on Alhydrogel^®^ (Alum) (Manufacturer) for administration with an aqueous formulation of the Toll-like receptor-4 agonist AP (activating protein) 10-701 (also known as glucopyranosyl lipid A). Clinical trials of the vaccine have been completed or are in progress in non-endemic and endemic communities [79]. An initial Phase 1a trial (https://clinicaltrials.gov/ct2/show/NCT02337855) of Sm-TSP-2/Alhydrogel^®^, given with or without AP 10-701, to *S. mansoni*-naïve healthy adults showed the vaccine formulations were safe and well tolerated and dose- and adjuvant-related increases in serum IgG against Sm-TSP-2 were observed [79]. A Phase 1b dose-escalation trial (https://clinicaltrials.gov/ct2/show/NCT03110757) of the safety, reactogenicity and immunogenicity of Sm-TSP-2/Alhydrogel^®^, with or without AP 10-701, in healthy exposed adults has been completed in a *S. mansoni*-endemic area of Brazil [79] but the results are not yet to hand. A Phase 1 dose-escalation safety and immunogenicity study in healthy exposed Ugandan adults followed by a Phase 2b trial in which a larger number of subjects will be enrolled to assess the impact of the Sm-TSP-2/Alhydrogel^®^ vaccine (± AP 10-701) on infection with *S. mansoni* are planned (https://clinicaltrials.gov/ct2/show/NCT03910972) with tentative completion in 2023. The impact of the vaccine on infection with *S. haematobium* will also be assessed although this will be exploratory given that potential cross-protection against this species is not yet proven. There is, however, some cause for optimism as recombinant versions of three *S. haematobium* extracellular vesicles (EV)-derived tetraspanins (including the *S. haematobium* homologue of Sm-TSP-2) were recently shown to induce protection, albeit moderate, in terms of reduced liver and intestinal eggs in a heterologous vaccine/challenge (*S. mansoni*) model of infection [80].

#### 5.1.4. Sm-p80

Sm-p80 (the large calcium activated neutral cysteine protease subunit of calpain) is expressed in all life cycle stages, and likely plays a key role in tegumental biogenesis and renewal in schistosomes [81]. Due to the major role it plays in schistosome survival, Sm-p80 has been assessed for vaccine efficacy using different vaccine approaches and formulations in three animal models (mouse, hamster and baboon) of infection and disease and shown to generate significant protection against *S. mansoni* challenge infections; moreover it evokes cross-species protection against *S. haematobium* and *S. japonicum* [71,72]. Importantly, Sm-p80-specific IgE antibodies were undetectable in exposed schistosome communities in Africa and South America Africa, thereby potentially minimising the risk of a hypersensitivity reaction against the vaccine [71,72]. In addition, Sm-p80 had a therapeutic effect in vaccinated baboons with reduced numbers of established worms and eggs retained in tissues, and decreased number of eggs excreted in faeces [71,72]. The Sm-p80/GLA-SE vaccine (*SchistoShield* ^®^) is planned to move forward (in 2020) to Phase 1 and 2 clinical trials in adults in endemic areas of sub-Saharan Africa (Burkina Faso and Madagascar) endemic for *S. mansoni* and *S. haematobium* [72,82].

### 5.2. Human Challenge Infection Model to Test Schistosomiasis Vaccines

In the search for novel vaccines (and drugs), an experimental human model for schistosomiasis could accelerate the development of these products. In a paradigm shift, the Roestenberg research group at Leiden University Medical Centre in the Netherlands is pioneering such an approach—the *S. mansoni* controlled human infection (CHI-S) model [64]. An effective CHI-S model could advance understanding of the protective immunity generated by vaccination and provide early protective efficacy data; this information can guide further clinical development of the most promising schistosomiasis vaccine candidates, thereby reducing the risk of costly downstream efficacy failure [64,83]. To date, the CHI-S work underway has resulted in the demanding production of the schistosome cercariae needed for human challenge that strictly adheres with all regulatory requirements and good manufacturing practice (GMP) principles. Safety is a critical issue in CHI trials; thus, to ensure volunteer safety during schistosome challenge, the Leiden group is using laboratory maintained single sex male cercariae of *S. mansoni*; these can infect human subjects and mature to adults but, as no eggs are produced, the egg-induced morbidity resulting from granuloma formation and fibrosis is avoided [64].

The findings (https://clinicaltrials.gov/ct2/show/NCT02755324) of the first *S. mansoni* CHI pilot trial in Leiden testing safety and dose finding in Dutch volunteers were recently reported [84]. Primary trial end-points were infectivity and recorded adverse effects. The authors reported an increase in 9 of 17 volunteers in adverse events associated with acute schistosomiasis (Katayama) syndrome [85] that was dose-related. All three volunteers of a high dose group, challenged with 30 cercariae, and two (of 11) of a medium dose group, challenged with 20 cercariae, reported severe adverse events. Worm-derived circulating anodic antigen (CAA), a highly sensitive primary infection endpoint biomarker, peaked in the sera of the majority (82%) of volunteers (at 3–10 weeks) following cercarial challenge. All volunteers seroconverted to IgG1 and IgM antibodies and exhibited worm-specific cytokine production by CD4+ T cells; all volunteers were cured following 1–2 doses of praziquantel. Overall, the experimental exposure to 20 male cercariae resulted in a detectable and well-tolerated *S. mansoni* infection in 82% of volunteers, an infection rate resembling that of other human infection models.

The CHI-S model provides a path for fast, proof-of-concept human trialling of schistosomiasis vaccines, and would be suitable for assessing the effectiveness of the currently available Sm14 and Sm-TSP-2 antigens; other vaccine candidates [65], either separately or in combination, could similarly be evaluated. The main limitation of the current model is the use of male schistosomes only so that vaccine targets more commonly expressed in females (e.g., Sm-p80) cannot be evaluated fully. A female worm infection model would thus be of value for dissecting mechanisms of action and sex-specificity of vaccines. The current Leiden CHI-S work provides a blueprint for future development of such a model using female cercariae only, thereby avoiding the morbidity that would otherwise follow. As pointed out by the Leiden team, despite the differences between chronic *S. mansoni* infection in the field and their controlled human *S. mansoni* infection, the CHI-S model provides the prospect of obtaining preliminary efficacy data on new candidate vaccines. Furthermore, by allowing selection of those most promising, the approach is highly cost-effective, given the prohibitive expense and large sample size required for current Phase 3 clinical testing.

## 6. Conclusions

Diseases caused by liver and blood flukes remain substantial threats to public health due to the high levels of morbidity they cause in many parts of the world. The negative economic consequences of *Fasciola* infections in ruminant livestock are also considerable. Currently, treatment is entirely dependent on a small number of effective drugs. Whereas vaccination is recognised as one of the most sustainable control options for many parasitic diseases, including those caused by the trematode flukes, development of protective and therapeutic vaccines against these pathogens has proven exceptionally difficult both scientifically and economically. The complexity of the life cycles of flukes and their capacity to evade host immunity add to the challenge of effective vaccine design, development and deployment. Immunity against these worms emphasises the Th1 type cellular response, the importance of antibodies and cytokines and the polarised type Th2 response and characteristic eosinophilia they cause.

Major recent advances in genomics, transcriptomics, proteomics, metabolomics, vaccinomics and other post-genomic technologies have aided new antigen discovery, but blood and liver flukes have largely resisted successful vaccine development efforts. It may be that the key immunological targets have still to be identified. Furthermore, the role of Th1 and Th2 responses and the balance between these two requires further exploration in trematode fluke infections and the possible induction of adverse auto-immune mediated mechanisms needs to be carefully considered in vaccine candidate selection. Many potential antigenic targets identified to date such as fatty acid binding proteins, glutathione-S-transferases and cathepsins, show genetic variation between target species possibly contributing to the reported inconsistent results between experiments. Moreover, many of these antigenic targets also have mammalian (including human) host homologues (paralogues, orthologues, etc), that may show variable levels of homology with those of their parasitic fluke counterparts. Additionally, it may be that vaccines targeted on fluke antigens that do not have a human homologue (e.g., excretory-secretory or tegumental-specific molecules) may deliver more promising results and reduce the possibility of potential induction of adverse immune responses such as autoimmunity.

It is a salient fact that the commercial development of vaccines for neglected tropical diseases in general has lagged behind other pathogens such as tuberculosis (TB), malaria, HIV and now SARS-CoV-2 coronavirus, and this poses an uphill battle. This is not only due to the complicated immunology associated with fluke infections but also the current limited level of funding that has resulted from not reaching vaccine development goals with sufficient speed [86]. Together with existing tools, blood and liver fluke vaccines tailored to fit into overall control program requirements would help achieve and sustain elimination. However, such vaccines would need to complement mass drug administration programs, without the addition of separate implementation costs, and be guided by mathematical modelling. Defining clear product development plans that reflect a vaccine strategy that complements existing control programs will be important in the path to developing effective fluke vaccines. In this respect, researchers working in the fluke vaccine space could consider using similar approaches to the burgeoning efforts currently underway with the COVID-19 vaccine pipeline [87], such as in vitro transcribed mRNA vaccines, a platform recently advocated for the development of neglected parasitic disease vaccines [88].

## Figures and Tables

**Table 1:** Efficacy of some single or combination vaccines tested against *Fasciola hepatica* (Modified from [15]).

Antigen	Source	Host	Schedule ^a^	Adjuvant	Efficacy ^b^ (%)
FhCL	Adult E/S	Cattle	10–500 μg × 3	FCA/FIA	38.2–69.5
			200 μg × 3		42.5
		Sheep	100 μg × 2		33
	Recombinant	Cattle	200 μg × 2	Montanide ^c^	47.2
				Montanide ^d^	49.2
		Goat	100 μg × 2	Quil A	0
					38.7
					0
				Montanide ^c^	0
	Mimotope	Goat	1 × 10^13^ pp	Quil A	46.9–79.5
FhCL1 + FhHb	Adult E/S	Cattle	200 μg × 3	FCA/FIA	51.9
FhCL2		Sheep	100 μg × 2	FCA/FIA	34
FhCL2 + FhHb		Cattle	200 μg × 3	FCA/FIA	72.4
				FCA/FIA	72.4
				FIA	11.2
				FCA/FIA	29
FhCL1 + FhCL2		Sheep	100 μg × 2	FCA/FIA	60
		Cattle	200 μg × 3	FCA/FIA	55
FhCL1 + FhCL2 + FhLAP Adult E/S	SOM	Sheep	100 μg × 2	FCA/FIA	79
FhCL1 + Prx + Sm14	Recombinant	Goat	100 μg × 2	Quil A	10.1
FhLAP	Adult Som	Sheep	100 μg × 2	FCA/FIA	89.6
	Recombinant	Rabbit	100 μg × 2	FCA/FIA	78
		Sheep	100 μg × 2	FCA/FIA	83.8
				Alum	86.7
				Adyuvac	74.4
				DEAE-D	49.8
				Ribi	49.5
FhPrx	Recombinant	Goat	100 μg × 2	Quil A	33.1
					33.1

FhCL1, *F. hepatica* cathepsin L1; FhCL2, *F. hepatica* cathepsin L2; FhHb, *F. hepatica* haemoglobin; FhLAP, *F. hepatica* leucine aminopeptidase; FhPrx, *F. hepatica* peroxiredoxin; Sm14, *Schistosoma mansoni* 14-kDa cytoplasmic fatty acid binding protein; Adult E/S or SOM, *F. hepatica* adult worm excreted/secreted or somatic products; Recombinant, recombinant protein; FCA/FIA, Freund’s complete/incomplete adjuvant; DEAE-D, Diethylaminoethyl-Dextran. ^a^ Vaccination dose (μg, micrograms; pp, phage particles number) and boosts number. ^b^ Reduction of worm numbers in vaccinated and infected animals, compared with infected and non-vaccinated animals. ^c^ Montanide ISA70VG. ^d^ Montanide ISA206VG. For further details of each vaccine trial, see Reference [15].

**Table 2:** Vaccine candidates tested against *Clonorchis sinensis* in the rat model of infection (Modified from [53]).

Vaccine Type	Protein	Worm Reduction Rate	Egg Reduction Rate
DNA (pcDNA3.1)	Cysteine proteinase	31.5%	15.7%
DNA (pcDNA3.1)	Fatty-acid-binding protein	40.9%	27.5%
*Bacillus subtilis* spore-based	Tegumental protein 22.3 kDa	44.7%	30.4%
Protein	Rho GTPase	60.5%	68.8%
Protein	14-3-3 epsilon	45.4%	37.9%
Protein	Paramyosin	54.3%	50.9%
DNA (pcDNA3.1)	Paramyosin	36.1%	38.8%
Protein	Enolase	56.3%	ND
Protein	Enolase	15.4%	ND
DNA (pcDNA3.1)	Enolase	37.4%	ND
*Bacillus subtilis* spore-based	Enolase	61.1%	80.7%
Protein	Cathepsin B cysteine protease (CB2)	41.0%	33.6%
Protein	Cathepsin B cysteine protease (CB3)	67.2%	57.7%

ND, not determined. For further details of each vaccine candidate trialled, see Reference [53].
